# Wild Poliovirus Importation, Central African Republic^1^

**DOI:** 10.3201/eid1906.121821

**Published:** 2013-06

**Authors:** Ionela Gouandjika-Vasilache, Arthur Mazitchi, Nicksy Gumede, Alexandre Manirakiza, Casimir Manenegu, Thomas D’Aquin Koyazegbe, Cara Burns

**Affiliations:** Institut Pasteur, Bangui, Central African Republic (I. Gouandjika-Vasilache, A. Mazitchi, A. Manirakiza);; National Institute for Communicable Diseases, Johannesburg, South Africa (N. Gumede);; World Health Organization, Bangui (C. Manenegu);; Ministère de la Santé, de la Population et de la lute contre le SIDA, Bangui (T. D’Aquin Koyazegbe);; Centers for Disease Control and Prevention, Atlanta, Georgia, USA (C. Burns)

**Keywords:** wild poliovirus, outbreak, importation, viruses, Central African Republic, vaccine, vaccination, polio, immunization, poliovirus, OPV, oral poliovirus vaccine, AFP, acute flaccid paralysis

**To the Editor:** Since the Global Polio Eradication Initiative was launched in 1988, indigenous transmission of wild poliovirus (WPV) has been interrupted in all countries except Afghanistan, Pakistan, and Nigeria (*1*). However, during 2003–2011, outbreaks resulting from importation of WPV occurred in 29 previously polio-free countries in Africa, including Central African Republic (CAR) (*1*–*3*). In 2011, 350 WPV cases were reported from 12 countries in Africa, a 47% decrease from the 657 cases reported by 12 countries in Africa in 2010 (*1*). 

In CAR, the last case of poliomyelitis caused by indigenous transmission of wild poliovirus was reported in 2000, but importation of WPV type 1 has been reported (*4*). We describe the importation of WPV1 and WPV3 into CAR during successive events in 2008, 2009, and 2011.

To investigate importation of WPV into CAR, we conducted a study using fecal samples collected from patients in CAR who had acute flaccid paralysis (AFP) during 2008–2011. The samples were analyzed for virus isolation, typing, and intratypic differentiation at the Regional Reference Laboratory for Polio, Institut Pasteur de Bangui, using World Health Organization (WHO) standard procedures (*5*). Isolated WPV strains were sent to the Centers for Disease Control and Prevention (Atlanta, Georgia, USA) or the National Institute for Communicable Diseases (Johannesburg, South Africa) for sequencing according to WHO guidelines (*6*–*8*). Cases were classified as laboratory confirmed or polio-compatible according to WHO recommendations; a polio-compatible case was defined as AFP for which stool samples were not adequate or a situation in which the patient was lost to follow up or had residual paralysis 60 days after testing.

Of 141 AFP cases from 2008, three, from Bangui, Ouham, and Ouaka districts, were laboratory confirmed as WPV1; this cluster was designated B2D1B (Figure). Sequencing results showed that the virus in this cluster belonged to the South Asia A (Indian) genotype, which was circulating in Angola and Democratic Republic of Congo at that time (Figure). 

**Figure:**
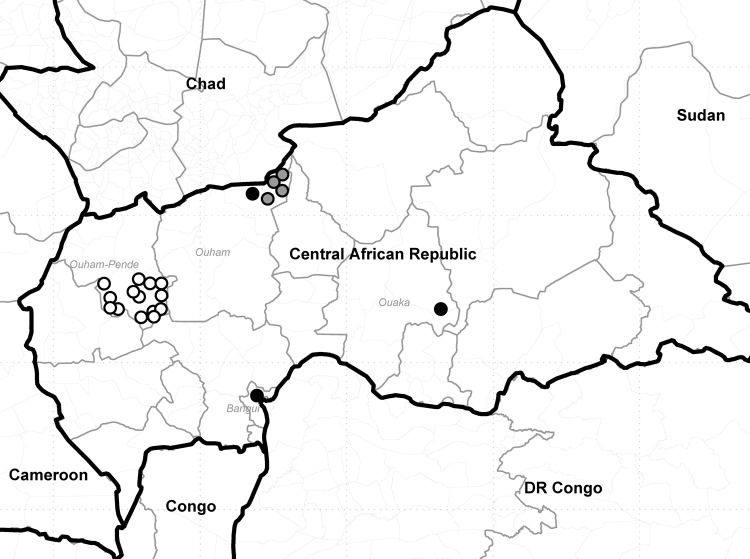
Clusters of polio cases caused by wild poliovirus importations, Central African Republic, 2008–2011. Each circle represents 1 case of acute flaccid paralysis confirmed as polio. Black circles, cluster B2D1B, 2008 poliovirus (PV) type 1 SOAS importation from Democratic Republic of Congo (DR Congo); white circles, cluster D2B2B1, 2009 PV3 WEAF-B importation from Nigeria and southern Chad; gray circles, cluster I6C2B4C1A2, 2011 PV1 WEAF-B importation from southern Chad.

Of 163 AFP cases from 2009, 14 in Ouham-Pende district were laboratory confirmed as WPV3; this cluster was designated D2B2B1. Sequencing results showed that the virus in this cluster belonged to West Africa B genotype, which was circulating in Nigeria and southern Chad at that time (Figure). 

Of 142 AFP cases from 2011, four in Ouham district were laboratory confirmed as WPV1; this cluster was designated I6C2B4C1A2. Sequencing results showed that the virus in this cluster belonged to West Africa B genotype, which was circulating in south Chad and Nigeria at the time (Figure).

The importation of wild poliovirus strains into CAR appeared to follow 3 different routes. In 2008, WPV1 originated from Democratic Republic of Congo and was first detected in the capital, Bangui, which is located in the southern part of the country. Two more cases were detected in 2 other districts, in the north (Ouham) and in the middle (Ouaka) of the country. During that year, 2 AFP cases were classified as polio-compatible; these cases originated from Haute Kotto and Ouham districts. In 2008, routine coverage of oral polio vaccine (OPV) was 45%, 33%, and 57% for Bangui and Sanitary Regions 3 and 4, respectively. (Sanitary regions are equivalent to provinces and have several districts under their jurisdiction; Bangui is considered a Sanitary Region containing 8 districts.) To interrupt wild poliovirus circulation, health authorities implemented 4 rounds of national immunization days, 2 using monovalent OPV (mOPV) type 1 and 2 using trivalent OPV; 1 local immunization day using mOPV1 was also instituted.

In 2009, WPV3 was imported from southern Chad to the Ouham-Pende district in CAR. This insecure district is difficult to access, but routine OPV coverage was reported as 61% for 2009, compared with the country’s official OPV coverage of 55%. The apparently higher coverage in areas of insecurity is likely the result of inaccurate target population estimates. Five additional AFP cases were classified as polio-compatible; these occurred in Ouham (1), Ouham Pende (2), Mambere Kadei (1), and Mbomou (1) districts. To interrupt wild poliovirus circulation, 8 supplementary immunization activities were organized.

The 2011 polio outbreak occurred in the district of Ouham and was caused by WPV1 poliovirus imported from southern Chad (Figure). Fourteen additional cases of AFP were classified as polio-compatible; these occurred in Ouham-Pende (6), Ouham (1), Ombela M’Poko (1), Kemo (1), Ouaka (1), Haute Kotto (2), and Mbomou (2) districts. Routine OPV coverage for this insecure and difficult-to-access region was 55% for 2010 and 58% for 2011.

CAR is one of the countries with the highest predicted risk for WPV circulation after importation (*9*). The 3 WPV importation events we report demonstrate that current immunization levels in this country are insufficient to guard against polio. High, sustained levels of routine OPV coverage in every district, supplemented by high-quality supplementary immunization activities, will help prevent future outbreaks. Surveillance standards must also be maintained to ensure the rapid detection of WPV importation, thus enabling timely response and containment.
